# Machine Learning Techniques in Blood Pressure Management During the Acute Phase of Ischemic Stroke

**DOI:** 10.3389/fneur.2021.743728

**Published:** 2022-02-14

**Authors:** Orit Mazza, Onn Shehory, Nirit Lev

**Affiliations:** ^1^Graduate School of Business Administration, Bar Ilan University, Ramat Gan, Israel; ^2^Lowenstein Rehabilitation Medical Center, Ra'anana, Israel; ^3^Neurology Department, Meir Medical Center, Kfar Saba, Israel; ^4^Sackler Faculty of Medicine, Tel-Aviv University, Tel-Aviv, Israel; ^5^Sagol School of Neuroscience, Tel-Aviv University, Tel-Aviv, Israel

**Keywords:** stroke, blood pressure, machine learning, hypertension, prediction

## Abstract

**Background and Purpose:**

Elevated blood pressure (BP) in acute ischemic stroke is common. A raised BP is related to mortality and disability, yet excessive BP lowering can be detrimental. The optimal BP management in acute ischemic stroke remains insufficient and relies on expert consensus statements. Permissive hypertension is recommended during the first 24-h after stroke onset, yet there is ongoing uncertainty regarding the most appropriate blood BP management in the acute phase of ischemic stroke. This study aims to develop a decision support tool for improving the management of extremely high BP during the first 24 h after acute ischemic stroke by using machine learning (ML) tools.

**Methods:**

This diagnostic accuracy study used retrospective data from MIMIC-III and eICU databases. Decision trees were constructed by a hierarchical binary recursive partitioning algorithm to predict the BP-lowering of 10–30% off the maximal value when antihypertensive treatment was given in patients with an extremely high BP (above 220/110 or 180/105 mmHg for patients receiving thrombolysis), according to the American Heart Association/American Stroke Association (AHA/ASA), the European Society of Cardiology, and the European Society of Hypertension (ESC/ESH) guidelines. Regression trees were used to predict the time-weighted average BP. Implementation of synthetic minority oversampling technique was used to balance the dataset according to different antihypertensive treatments. The model performance of the decision tree was compared to the performance of neural networks, random forest, and logistic regression models.

**Results:**

In total, 7,265 acute ischemic stroke patients were identified. Diastolic BP (DBP) is the main variable for predicting BP reduction in the first 24 h after a stroke. For patients receiving thrombolysis with DBP <120 mmHg, Labetalol and Amlodipine are effective treatments. Above DBP of 120 mmHg, Amlodipine, Lisinopril, and Nicardipine are the most effective treatments. However, successful treatment depends on avoiding hyponatremia and on kidney functions.

**Conclusion:**

This is the first study to address BP management in the acute phase of ischemic stroke using ML techniques. The results indicate that the treatment choice should be adjusted to different clinical and BP parameters, thus, providing a better decision-making approach.

## Introduction

Machine learning (ML) applications in healthcare have significant potential for improving clinical decision-making diagnoses, treatment effectiveness, and healthcare management, including lowering the costs for both healthcare providers and patients (1). ML applications for Knowledge Discovery in Databases (KDD) have been used for more than two decades and are useful for discovering information and extracting knowledge from data and reflect a multi-step process that involves thorough data preparation, pattern searching, and knowledge evaluation (2). The use of ML to extract non-trivial and previously unknown useful information from data may be most beneficial for physicians in areas where the level of evidence or class of recommendation is low and will increase the likelihood of the physicians adopting them (3). The use of ML in clinical research to predict a particular clinical outcome is useful because it has the potential to outperform the best clinical knowledge obtained by current traditional medical research. In this study, we applied the KDD process by using ML techniques to conduct a robust interrogation to identify predictors of blood pressure (BP) management after acute ischemic stroke, thus, having the potential to aid clinicians in improving treatment regimens.

An elevation in BP is common in the acute phase of a stroke and occurs early at the time of arrival to the emergency room. In two-thirds of the patients, elevated BP was transient and resolved within 2 weeks from symptom onset (4). Observational studies have shown that elevated BP during ischemic stroke onset is prognostically associated with an increased risk of early adverse events and mortality. However, acute and aggressive BP lowering within 24 h of stroke onset could also jeopardize the outcome (5). Both elevated and low BP are independent factors that predict poor outcomes among patients with acute ischemic stroke and present a U-shaped relationship between BP and death or disability (6, 7).

High BP in acute stroke can decrease blood perfusion to areas of ischemic brain tissue, which, in turn, can cause neurological damage (8). An extremely high BP can result in intracerebral bleeding and hypertensive emergencies, including renal failure, ischemic heart disease, and pulmonary edema (9). In patients who received thrombolytic treatment, studies concur that there is a strong association between high BP and worse clinical outcomes, including death, disability, and hemorrhagic transformation (7, 10). The AHA/ASA and ESC/ESH guidelines recommend lowering the BP below 180/105 mmHg in patients receiving thrombolysis in the first 24 h after acute stroke, a strong class of recommendation (class I). In patients not receiving thrombolysis, a clinical judgment is defined as whether to treat hypertension when it exceeds 220/120 mmHg, a weak class of recommendation (class II-b) (11, 12). There is no firm evidence regarding BP management in patients with acute ischemic stroke with a BP lower than 220/120 mmHg, who did not receive thrombolysis (12). The specific interval for BP reduction is not well-established, and the current approach of lowering BP by 15% is considered reasonable by a consensus expert opinion (11, 12). The current recommended approach by the AHA/ASA is to treat with labetalol, nicardipine, or clevidipine when systolic blood pressure (SBP) is over 180–230 mmHg or diastolic BP (DBP) is over 105–120 mmHg. If DBP exceeds 140 mmHg or is not controlled by these treatments, sodium nitroprusside is recommended. However, these recommendations are not based on a strong class of recommendations (11, 13).

There are therapeutic strategies for elevated BP that are not included in the current acute stroke guidelines. In most hypertensive emergencies, intravenous (IV) drug administration is considered, although oral therapy with ACEI/ARBs or beta-blockers is effective in the acute setting of hypertensive emergency because of the activation of the renin system. Besides the medications mentioned above for BP lowering in acute ischemic stroke, other treatment options are utilized in various hypertensive emergencies including metoprolol, esmolol, nitroglycerine, clonidine, and enalaprilat. The duration of action of these treatments ranges from several minutes to several hours and enables dose adjustment according to clinical judgment (12). The use of these medications in the treatment of acute ischemic stroke is required.

Few randomized clinical trials have examined the impact of BP reduction immediately after acute stroke with antihypertensive agents (14, 15). The effects of continuous antihypertensive treatment, in previously known patients who were hypertensive after acute stroke in the Continue Or Stop post-Stroke Antihypertensives Collaborative Study (COSSACS), showed a statistically significant reduction of 13/8 mmHg in BP at 2 weeks in the continuing group compared to the stop group, and no differences emerged between the groups in rates of serious adverse events, 6-month mortality, or major cardiovascular events (14). However, the aforementioned study had inherent limitations due to the complex clinical situation. It was not placebo-controlled, and there was a multiplicity of pre-existing antihypertensive treatments (14). The China Antihypertensive Trial in Acute Ischemic Stroke (CATIS), a randomized clinical trial, compared patients who received antihypertensive treatment to those who discontinued all antihypertensive medications during hospitalization. The treatment aimed to lower SBP by 10–25% within the first 24 h. The primary outcome of death within 14 days after randomization and major disability at 14 days or hospital discharge did not differ between the groups. However, early antihypertensive therapy was associated with a lower rate of 3-month recurrent stroke among patients with a history of hypertension (15).

Several randomized clinical trials have examined the use of specific antihypertensive agents (16, 17). The Controlling Hypertension and Hypotension Immediately Post Stroke (CHHIPS) randomized controlled trial investigated the effect of BP reduction with labetalol and lisinopril vs. placebo in patients with SBP > 160 mmHg. The SBP reduction within the first 24 h was higher in both treatment groups (16). The Intravenous Nimodipine West European Stroke Trial (INWEST) showed a significant decrease in SBP and DBP with nimodipine treatment vs. placebo in the first 48 h (18). Furthermore, several randomized trials have examined the effects of angiotensin receptor blockers (ARBs) on BP reduction in the acute phase of stroke and observed a modest reduction in BP of up to 10/6 mmHg in the treatment group vs. the placebo group (17, 19, 20).

In many clinical trials evaluating BP-lowering, markedly elevated BP ranges (usually > 220/120 mmHg) were excluded (14–16). However, the guidelines concern the treatment of severe hypertension. In addition, no solid data are available to guide the selection of antihypertensive treatment. Accordingly, the main objective of this research was to develop a decision support tool for improving the management of extremely high BP during the first 24 h after acute ischemic stroke by using ML techniques. To date, no published study has used ML techniques to predict BP management in the acute phase of ischemic stroke.

## Methods

The source codes for the analyses can be found at: https://github.com/OritMazza/BP_managment_AIS.

The MIMIC Code Repository is open source and available online. It was used with minor changes for the variable extraction tasks and can be found at the following link: https://github.com/MIT-LCP/mimic-code.

### MIMIC-III and eICU Collaborative Databases

We used two large, public, and freely accessible intensive care unit (ICU) databases of de-identified patients: the eICU Collaborative Research Database (eICU-CRD) and the Medical Information Mart for Intensive Care III (MIMIC-III) database in a diagnostic accuracy study based on retrospective multicenter data. Adult patients admitted to critical care units at the Beth Israel Deaconess Medical Center between 2001 and 2012 with acute ischemic strokes were selected from the MIMIC-III v1.4 Critical Care Database (21). Similar cohorts of adult patients who were admitted to critical care units across 208 hospitals throughout the United States during 2014–2015 were recruited from the eICU Collaborative Research Database v2.0 (22). The two datasets are independent because the hospital source of MIMIC-III is not included in the eICU program (22). The databases were accessed through the Google BigQuery platform, a relational database management system, and the data were extracted from the two reference databases using SQL queries (23).

### Cohort Selection

The cohort included adult patients aged 18–88. Older patients were excluded because of the de-identification process of the MIMIC-III and eICU databases, which obscured the identities of patients above 89 years old to comply with the Health Insurance Portability and Accountability Act (HIPAA) regulations (21, 22).

The ICD-9 codes were identified for acute ischemic stroke as follows: 433 (occlusion and stenosis of precerebral arteries), 434 (occlusion of cerebral arteries), and 436 (acute but ill-defined cerebrovascular disease). The ICD-9 codes were selected according to their highly predictive values for actual cases of acute ischemic stroke, as previously described (24). The 433.x0 ICD-9 code was excluded because of the very low PPV in several studies, and 433.x1 was included to select more cases of acute ischemic stroke with little impact on PPV (25). Patients with ischemic stroke who received IV-tPA were identified from the MIMIC-III database according to ICD-9 procedure code 99.1, and those with endovascular treatment (EVT) were identified by ICD-9 code 39.74 (26). Because of the differences between the databases regarding the thrombolytic treatment identification process, patients with acute ischemic stroke who received IV-tPA were identified from the eICU database by using the *treatment* table with the *treatmentString* variable and the keyword “thrombolytics.” Table I in the Supplemental Material provides a list of the selected ICD-9 codes and the number of diagnoses according to different subgroups of acute ischemic stroke codes.

A time window of 24 h from hospital admission was selected for patients who did not receive EVT/tPA. According to guidelines, tPA is administered at the hospital up to 3–4.5 h from ischemic stroke symptom onset and, therefore, is usually administered at a time window of 24 h from admission to the hospital (11). A time window of 24 h after receiving tPA treatment was selected for patients who received thrombolytic treatment. Therefore, the cohort also included patients who underwent acute ischemic stroke at the hospital and received tPA treatment.

**Figure 1:**
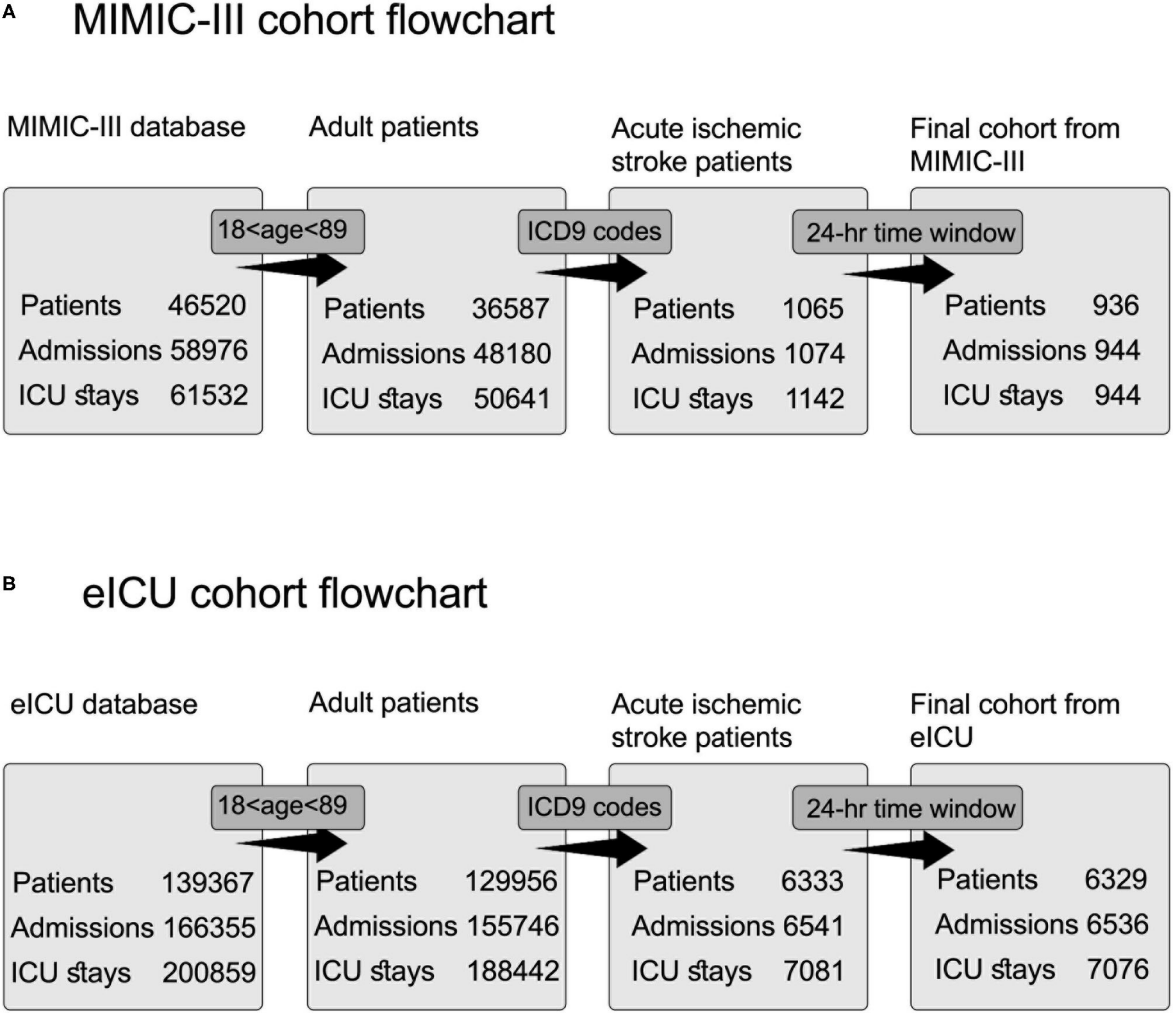
Cohort selection flowchart for acute ischemic stroke patients from two databases. Each step includes a distinct number of patients, admissions, and ICU stays. The query process consists of three steps: selection according to age inclusion criteria, extraction according to ICD9 codes, and extraction according to the time window of 24 h. **(A)** Flowchart for cohort selection from MIMIC-III database. **(B)** Flowchart for cohort selection from eICU database.

Figure 1A shows the MIMIC-III cohort flowchart. In the MIMIC-III database, exclusion by ICD-9 code sequence as the first or second diagnosis enables accurate selection of patients with acute ischemic stroke during the querying process and prevents the selection of admissions of the same patients with other primary clinical conditions. Left joining with the icustay table (*icustay_id*) for each patient resulted in 1,142 ICU admissions (*icustay_id*) and 1,061 hospital admissions (*hadm_id*). The extraction of the selected time window of 24 h from hospital admission (*admittime*) to the ICU admission time (*intime*) resulted in a unique ICU stay for each patient in the selected cohort.

Figure 1B shows the eICU cohort flowchart. Patients selected according to their ICD-9 codes for acute ischemic stroke were documented as active problems during the ICU stay using the diagnosis table.

### Knowledge Discovery in Databases and the ML Approach

**Figure 2:**
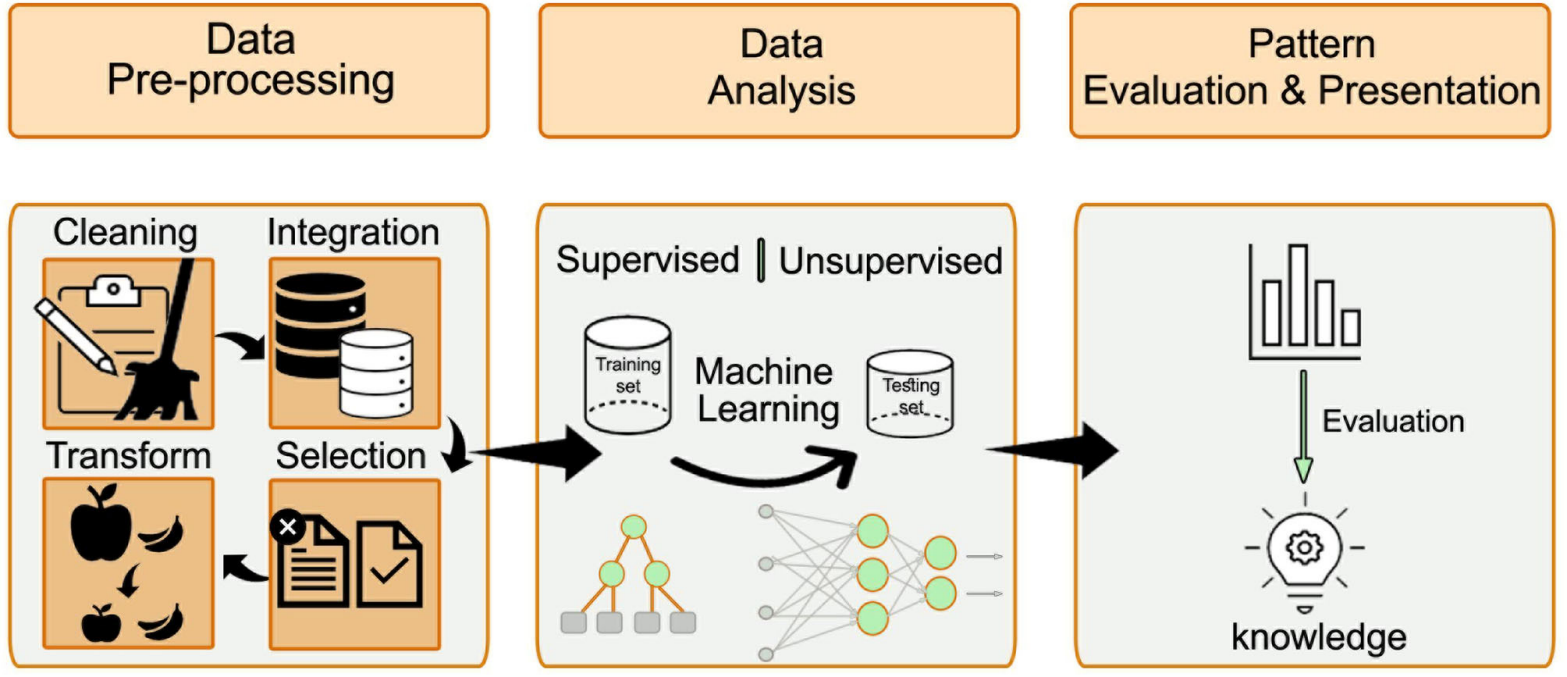
The major steps of the KDD process: data pre-processing, data analysis, and patterns evaluation. The pre-processing step includes data cleaning, data integration, data selection, and data transformation. After preparing the data, the ML approach is selected (either supervised or unsupervised learning). The processed data is divided into training and validation datasets. Different algorithms are compared to find the best model that fits the prediction task.

The KDD process is divided into three major steps, which we followed in our study and are illustrated in Figure 2. The first step is the data pre-processing step, which includes data cleaning, data integration, data selection, and data transformation. Data cleaning involves identifying and handling corrupt, incorrect, inaccurate, and irrelevant data, as well as the missing values. Data integration entails combining data from different sources to generate a unified view and ensuring that the same variables are within the same scale, and each variable has a single meaning. In addition, data are integrated from several file formats, for example, video or audio with text or tables. Data selection entails choosing the relevant data using different methods, such as feature selection or principal component analysis. Data transformation aims to normalize or standardize the independent variables to avoid bias resulting from using variables with a different range of values.

The second step after preparing the data is the use of ML techniques. According to the research question and the data available, the ML approach is selected (either supervised or unsupervised learning). When the predicted outcome is well-defined and can be labeled, supervised learning is preferred. The selection of specific algorithms is guided by the dependent variable of the prediction task. When the dependent variable is of continuous type, a regression algorithm is typically selected. When the dependent variable is of categorical type, a classification algorithm is typically selected. Some algorithm types, for example, random forest and artificial neural networks, fit both classification, and regression problems. An unsupervised learning approach is considered when the prediction task is unknown. In such cases, pattern identification and recognition techniques can be applied.

The processed data were divided into training and validation datasets. The training dataset is used to produce a trained and fitted model that generalizes well to unknown data. When a large amount of data is available, a sample of the original dataset—the validation dataset—is held back from the training model and is used to evaluate the model performance to obtain an unbiased result of the model's effectiveness. Typically, in this step, different algorithms are compared to identify the best model that fits the prediction task. The final step entails the evaluation of the patterns and their presentation. This step is essential to ensure that useful knowledge is derived from the data.

### Primary Outcome

**Figure 3:**
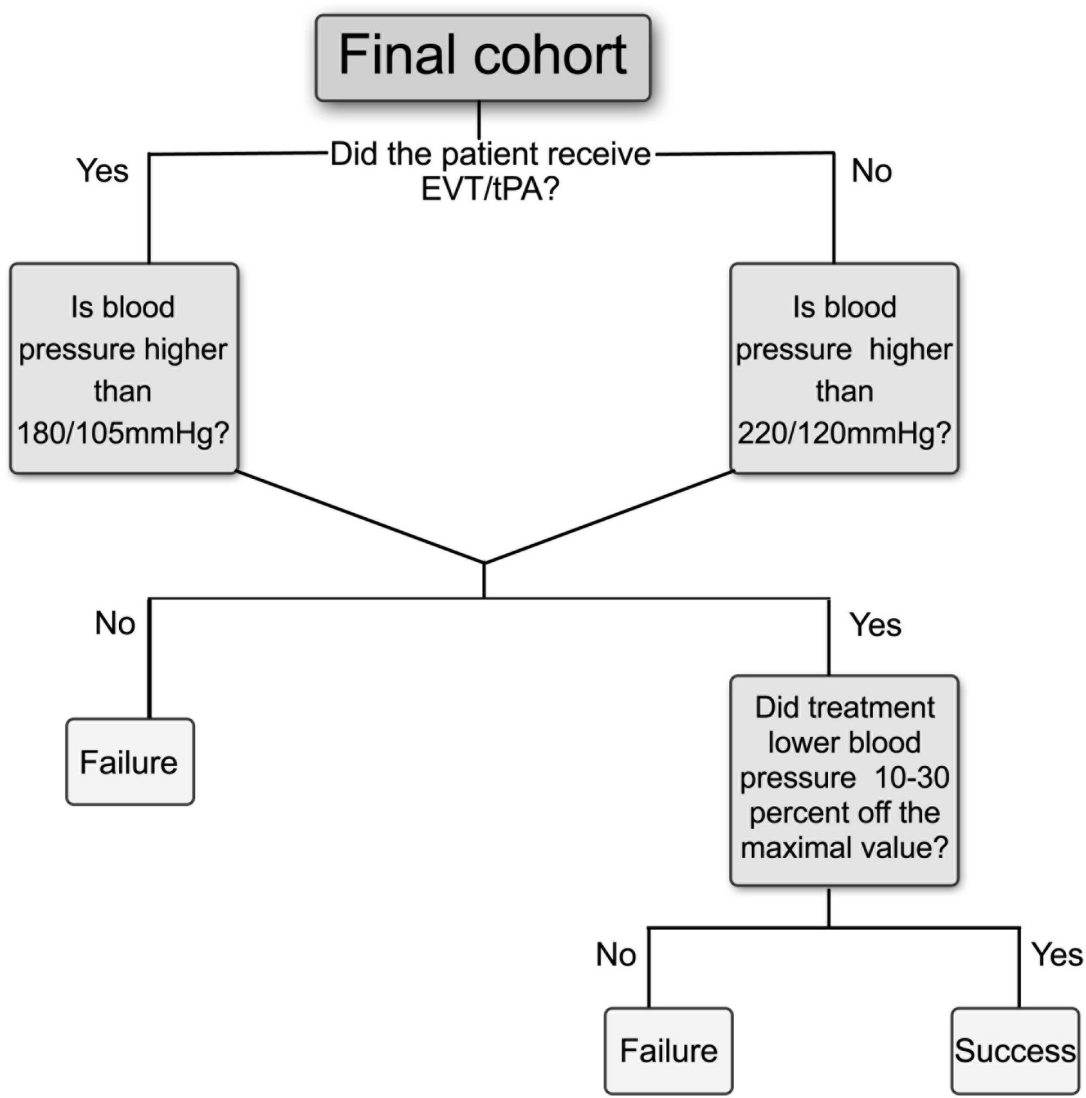
Flowchart for primary outcome criteria to assess BP management in the 24 h after acute ischemic stroke. Positive outcomes are considered as “Success” if initiation of therapy was given according to guidelines thresholds and if the average BP was decreased at least 10% and up to 30% off the maximum value during the selected time window, otherwise determined as “Failure.” Different thresholds for starting antihypertensive treatment were determined according to whether patients received EVT or tPA, or not. EVT, Endovascular treatment; tPA, tissue plasminogen activator.

Figure 3 shows the flowchart of how the primary outcome was determined and which criteria were selected for the assessment of BP management. A BP management was determined successful when BP treatment was administered according to the AHA/ASA and ESC/ESH guidelines and resulted in a BP reduction of 10–30% of the maximum value that was measured during the time window of 24 h. Our scheme model, as represented in Figure 3, was built in accordance with the guideline recommendations that antihypertensive treatment should be restricted to high BP and, thus, created decision rules according to different BP levels. The threshold for starting antihypertensive treatment after acute ischemic stroke was set at > 180/105 mmHg for patients who received EVT/tPA and > 220/120 mmHg for non-tPA patients (11). There is no evidence regarding the exact interval for BP reduction when it is markedly elevated and exceeds the recommended threshold. However, several studies have suggested that this interval is safe or associated with good clinical outcomes in patients with acute ischemic stroke (15, 27).

Further evaluation of the time-weighted average (TWA) of SBP and DBP was calculated and used as a continuous outcome variable in the prediction of algorithms. The TWA provides a less biased and more accurate estimation of BP than a simple average (28) and can be used to evaluate a BP after admission for acute stroke (10, 29).

### Dataset Pre-processing

The MIMIC-III (v1.4) relational database contains 26 different tables relating to unique patients, unique admissions to hospitals, and unique admissions to ICUs (21). The eICU Collaborative Database (v2.0) contains 31 tables concerning each ICU stay (22). We extracted 91 variables from seven different tables in the MIMIC-III database (*diagnoses_icd, patients, admissions, icustays, chartevents, labevents*, and *prescriptions*) and eight different tables in the eICU database (diagnosis*, treatment, vitalaperiodic, apacheapsvar, vitalperiodic, medication, lab*, and *patient*).

We extracted 91 variables as possible attributes for prediction algorithms. The variables were divided into four groups: demographic, hemodynamic and vital signs, laboratory results, and comorbidities. We included 91 out of 103 predictors used by Wang et al.'s pipeline for the MIMIC-III database (30). Because of the large number of missing values, unlike Wang et al. variables that indicated mechanical ventilation were excluded, and instead, we used the covariate of persistence or absence of mechanical ventilation, whether one or more of the following variables were present in the MIMIC-III database: ventilator type, ventilator mode, respiratory pressure, tidal volume, minute volume, inspiratory pressure, plateau pressure, positive end-respiratory pressure (PEEP), airway pressure release ventilation (APRV), high-pressure relief, pressure-controlled ventilation (PCV) levels, time-cycled pressure-controlled ventilation (TCPCV), and pressure support ventilation (PSV) levels. In contrast to the MIMIC-III, the eICU database has the *vent* variable in table *apacheApsVar*, which contains information on whether the patient was ventilated at the time of the worst respiratory rate. Similar to the mechanical ventilation variable, we used the absence or persistence of central venous pressure (CVP), cardiac output (CO), and cardiac index (CI) as covariates in both databases because of their high prevalence of missing values, and the importance of including them as indicators of the need for more intensive monitoring care.

Comorbidities in the MIMIC-III and eICU databases were selected according to Quan et al. and enhanced the ICD9-CM coding algorithm, which is also provided in the MIMIC-III repository (30, 31). We used the U.S. National Library of Medicine RxMix application programming interface (API) (https://mor.nlm.nih.gov/RxMix/) to identify the members of each antihypertensive drug class. Then, we extracted prescriptions for antihypertensive medications in the first 24 h in the ICU or from the time of onset of the tPA treatment. The output for each drug class was stored as a binary result, namely, whether the medication from the specific drug class was administered (1) or not (0). We included 106 antihypertensive medications, which were divided into eight different drug classes. Table 1 shows the drugs that were used in the selected time window and the prevalence of use in the selected cohort.

**Table 1:** Drugs and classes of antihypertensive medications.

**Class**	**Drugs**	**Routh of administration**	**Positive observations (*n* = 8,025)**	**%**
CCBs	Amlodipine (38.1%) Diltiazem (21.1%) Nicardipine (35%) Nifedipine (0.4%) Nimodipine (0.9%) Verapamil (0.2%) Combinations (4.3%)	Enteral Enteral IV±PRN IV Enteral Enteral Enteral	683	5.2
Beta-blockers	Atenolol (0.8%) Carvedilol (4.8%) Esmolol (0.04%) Labetalol (45.62%) Metoprolol (33.6%) Nadolol (0.04%) Combinations (15.1%)	Enteral Enteral IV IV±PRN Enteral/IV±PRN Enteral	2,374	29.2
ACE-inhibitors	Captopril (1.7%) Enalapril (1.2%) Lisinopril (96.8%) Quinapril (0.3%)	Enteral Enteral/IV Enteral Enteral	408	5
ARBs	Losartan (90%) Valsartan (10%)	Enteral Enteral	40	0.5
Diuretics	Bumetanide (0.9%) Furosemide (84.8%) Hydrochlorothiazide (8.2%) Spironolactone (4.2%) Combinations (4.1%)	Enteral/IV Enteral/IV Enteral Enteral	655	8
Direct vasodilators	Hydralazine (100%)	Enteral/IV±PRN	1,163	14.4
Sympatholytic agent	Clonidine (100%)	Enteral/TD	79	1
Other	Nitroprusside (100%)	IV±PRN	34	0.4

*CCBs, Calcium channels blockers; ACE, Angiotensin-converting enzyme; ARBs, Angiotensin receptor blockers. Enteral includes the following routes of administrations: PO (Per os), NG (nasogastric), and PZ (Per Zonda), PEG (Percutaneous endoscopic gastrostomy). IV: Intravenous. PRN (Pro-re-nata) refers to the administration of prescribed medication as needed or as the situation arises according to physician instructions. TD, Transdermal*.

The data cleaning process included excluding variables, which were marked as errors by physicians using the error variable of the *chartevents* table. To ensure that a single maximum value is not subjected to outliers or erroneous measurements, we added a variable that counts the number of times that the BP values elevated beyond the thresholds and excluded the BP values that were only elevated once in the selected time window. Different *itemids* for laboratory results or vital signs were grouped according to their clinical taxonomy to reduce missingness and duplicate measures, similar to other studies (30). All variable units with more than one *itemid* were examined. Height, weight, and temperature with different measuring units were standardized to meters, kilograms, and degrees Celsius, respectively. We used the minimum, maximum, and average of the numerical values of the time series variables. A similar approach was used by Purushotham et al. and Wang et al. to aggregate time series variables using average values or summations at their selected time windows (30, 32). The same variables were selected from the eICU database with the same measuring units as those in the MIMIC-III database. Additionally, the eICU time series variables were treated the same as in the MIMIC-III database.

Variables with more than 20% of missing values were excluded from the dataset to avoid bias in the standard deviation. We could not include two important risk factors as predictors, namely, lipidogram and smoking status, because of insufficient data. Missing values were replaced with average values.

Numerical variables were normalized in the range of 0–1 (0 ≤ z(i) ≤ 1) for better performance according to the following formula:


$$\begin{array}{l} Z\left(i\right)=\frac{x\left(i\right)-min\left(x\right)}{\left(x\right)\;-min\left(x\right)} \end{array}$$

where Z(i) is the normalized variable, and x(i) is the original variable at index i=1, 2, …, 91.

We examined the interactions between the independent variables and excluded the highly correlated predictors to avoid multicollinearity problems. Then, to successfully apply our data mining techniques to our dataset, we had to decrease the number of input variables to simplify the results and provide a better understanding and visualization (33). We selected a subset of our original variables using the feature selection method, which does not transform the variables and selects them from the existing dataset (34). The results were produced using the freely available software R, version 3.6.3 (35). Bidirectional stepwise elimination was used with the *step ()* function from the MASS package in R (36). The training dataset included two-thirds of the sample, and one-third was used to test the dataset. A description of the variables can be found in Table 2 in the Supplementary Appendix.

### Models

Decision tree models are useful for decision-making and are prevalent in healthcare research and, thus, were selected as our primary approach for evaluating the data (37). We compared the performance of this model with logistic regression, random forest, and neural network algorithms. The performance of the different models on the classification task was measured by the *confusionMatrix ()* function using the R package *caret* (38). We used the mean accuracy, kappa, and F-score values of the validated dataset to assess the overall performance of the classification models. To predict the regression task, the root means square error (RMSE) and the mean absolute error (MAE) were used to evaluate the performance of the regression tree model.

### Balanced Dataset According to Different Antihypertensive Treatments

For comparison purposes, we used the technique of treating the imbalanced classification problems to lower biased predictions that resulted from an imbalanced dataset that includes a different number of medications that were used. For example, in the original datasets, 239 patients received nicardipine, while 1,082 patients received labetalol in the first 24 h, as presented in Table 1. The differences between the absolute number of medications that were used might result from specific suggestions in guidelines for the acute lowering of BP in the treatment time window or from the inherent heterogeneity of a multicenter study, which may stem from differences in practice, including differences in maximum doses and titration protocols between different centers. This may partly explain the differences in the probability of a successful BP lowering between different medications. We were unable to include the doses that were used because the doses were recorded differently at different centers and their aggregation would have resulted in inaccurate doses. To reduce this bias, we created a new, balanced synthetic dataset based on the different medications that were used. In each iteration, new synthetic data were created for a specific treatment. To balance the data, the medications that were used as independent variables in the original dataset were used as dependent variables to create the balanced synthetic data. After creating balanced synthetic observations for each treatment, all the new synthetic observations were aggregated to a final balanced dataset. In the final balanced dataset, we included the same absolute number of eight medications (the most prevalent in use in this dataset and have at least 30 records in the dataset), specifically labetalol, metoprolol, carvedilol, hydralazine, lisinopril, furosemide, amlodipine, and nicardipine. The final balanced dataset included 300 observations for each treatment, resulting in a total of 2,400 observations for eight different treatments that were detected. In the balanced dataset, we only included the patients who were treated with a single antihypertensive treatment and excluded all the observations of patients who were treated with more than one drug. Apart from the balanced observations of patients who were treated with one antihypertensive treatment, we included patients who were not treated with antihypertensive drugs in the first 24-h window. The balanced dataset was prepossessed in a manner similar to that of the original dataset. The original dataset was divided into training and testing datasets at a 2:1 ratio. The test set was left aside and saved to examine the performance of the balanced dataset on the original test set that reflects real-world data. We used the Random Over Sampling Example (ROSE) package to generate a new synthetic, balanced dataset for each treatment based on sampling methods and the smoothed bootstrap approach. The new synthetic data are generated from the conditional kernel density. We used the *ovun.sample ()* function, which enables simultaneous oversampling and undersampling (39, 40).

## Results

The final cohort included 7,265 patients with acute ischemic stroke, 7,470 admissions to hospitals, and 8,020 ICU stays. Among all ICU stays, 1,579 (20%) were treated with tPA or EVT. Of the ICU-stay cases, 694 (10%) met the criteria for “Success” of the primary outcome. Table 2 shows the characteristics of the study population.

**Table 2:** Characteristics of the study population.

**Patient characteristic**	**Prevalence, (%) or Mean ±SD**
**Sex**	
Women	46.8
Age, y	66.5 ± 13.6
18–29 (%)	1.5
30–49 (%)	9.8
50–69 (%)	42.3
70–89 (%)	46.6
**Ethnicity**	
Caucasian (%)	74.4
African	12
American (%)
Hispanic (%)	4.5
Asian (%)	2
Other (%)	7.1
**Selected comorbidities (%)**	
Hypertension (%)	27.4
Cardiac arrhythmias (%)	17.2
Diabetes uncomplicated (%)	7.4
Diabetes complicated (%)	0.5
Renal failure (%)	7.8
Congestive heart failure (%)	6.7
Chronic pulmonary disease (%)	7.2
Hypothyroidism (%)	3
Alcohol abuse (%)	1.7
Received endovascular or thrombolytic treatment (%)	19.7
**Antihypertensive drug class**	
Beta-blockers (%)	29.2
Direct vasodilators (%)	14.4
CCBs (%)	8.4
Diuretics (%)	8
ACE-I (%)	5
Sympatholtics agent (%)	1
ARBs (%)	0.5
Nitroprusside (%)	0.4
**Number of antihypertensives drug classes**	
0 (%)	60.9
1 (%)	19.4
2 (%)	13.7
3 (%)	4.6
4 (%)	1.3
5 (%)	0.2
Time-weighted average SBP (mmHg)	133.6 ± 20.1
Time-weighted average DBP (mmHg)	70.3 ± 18.2

*SD, Standard deviation; CCBs, Calcium channels blockers; ACE-I, Angiotensin-converting enzyme inhibitor; ARBs, Angiotensin receptor blockers*.

### Decision Tree Model

The tree is constructed by a hierarchical binary recursive partitioning algorithm, which enables the visual representation of statistically significant results as a tree. Unlike popular techniques for building trees, CART and C4.5, the conditional interference tree (Ctree) method examines whether the covariates and the response variable are statistically significant (*p* < 0.05) and have a better handle on the overfitting problem and selection bias toward covariates with many possible splits (41). The Ctree was implemented using the *ctree ()* function with the R package *party*, which is useful for predicting both the categorical outcome (classification trees) and continuous outcomes (regression trees). The minimum criterion for each split was selected as *p* < 0.05, and the input variable with the smallest *p*-value was used for the next division (41).

**Figure 4:**
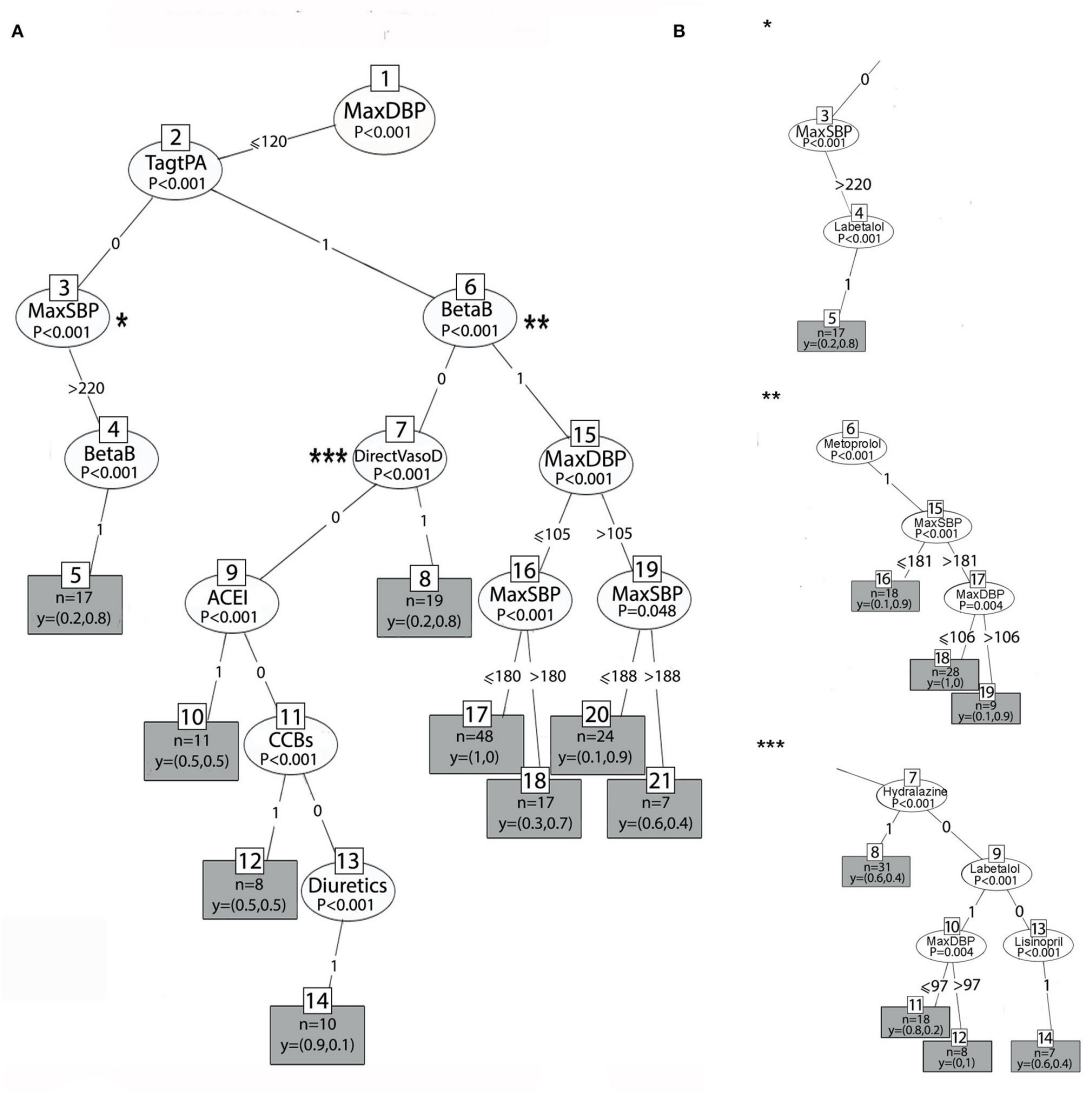
**(A)** The right branch of the classification tree. Each split is represented by a node. The first split is the root node, and the others are the intermediate nodes. For each node, the Bonferroni-adjusted *P*-values are given. Each rectangle is a terminal node which is showing the probability distribution of occurrence over the two classes of the primary outcome and includes the number of observations associated with the specific node result that files the decision claims. **(B)** Subtrees represent the most prominent medication that lowers BP to the selected interval from each drug class by astricts.

**Figure 5:**
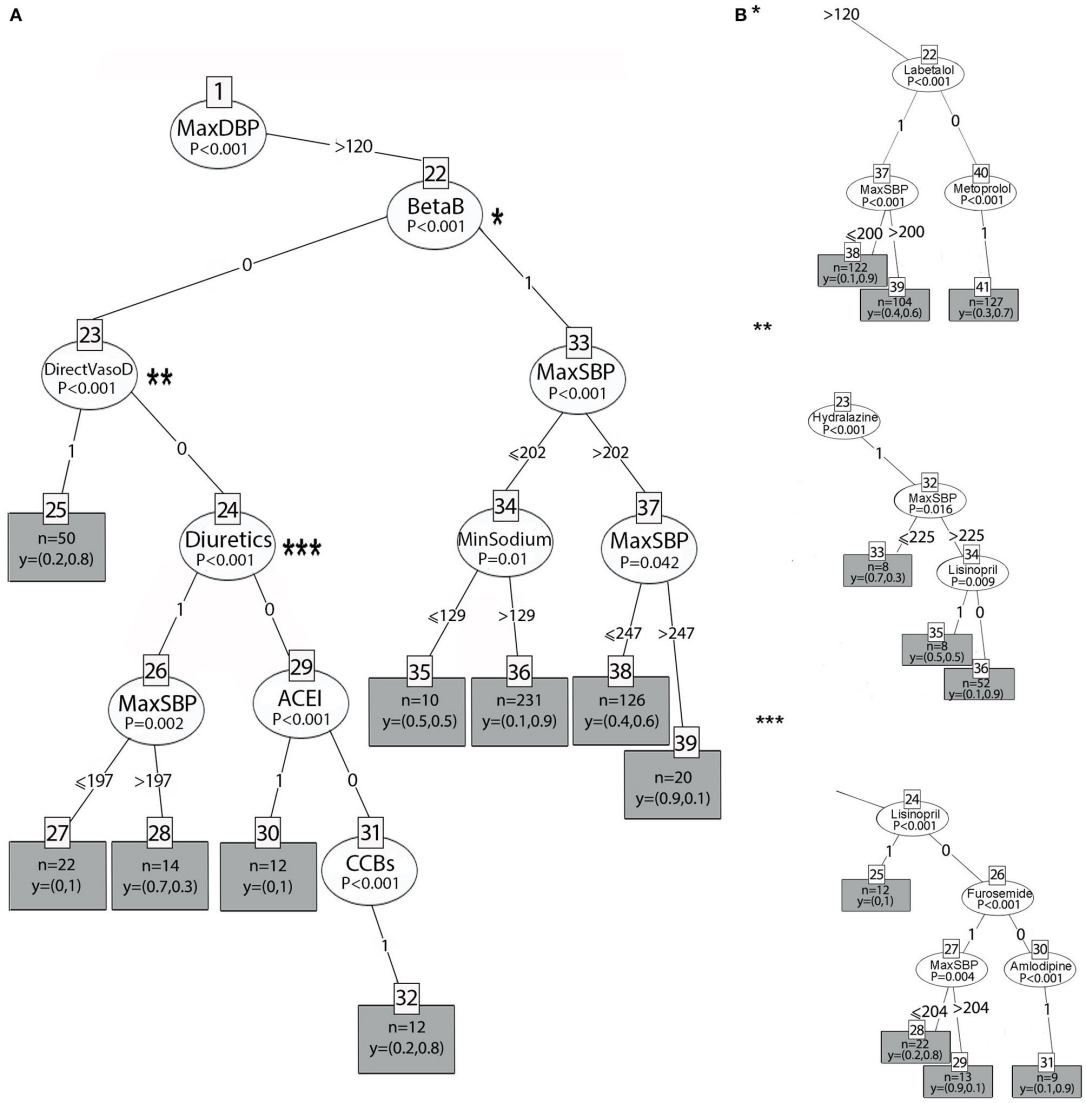
**(A)** The left branch of the classification tree. **(B)** Subtrees are represented by asterisks (*, **, ***) and show the most prominent medication that lowers BP to the selected interval.

Figures 4A, 5A show the two main branches of the conditional interference tree. A variation of this tree is represented in Figures 4B, 5B as subtrees. In Figures 4B, 5B, the drug classes were replaced with the specific medications that were used in the first 24 h, as shown in Table 1. Additional information regarding the specific medications that reduce BP to the selected interval provides clinicians with more information regarding which medication is most effective for BP reduction from every drug class. For each branch, three subtrees were constructed and are represented by asterisks (^*^, ^**^, and ^***^). The different drug classes were replaced by the drug that induced the greatest reduction, as shown in Figures 4B, 5B.

In Figures 4A, 5A, at the top of the tree, the strongest associated variable (*p* < 0.001) is the maximum DBP. The splitting criteria for the first node are based on whether the maximum DBP that was observed in the 24-h time window was above 120 mmHg (left branch) or equal to or below 120 mmHg (right branch). Figure 4A shows the right branch of the tree. The second split is based on whether the patient received tPA or EVT, as follows: TagtPA = 1 for “received” and TagtPA = 0 for “not received” (node 2). On the right side for patients who received EVT or tPA, when the DBP was between 105 and 120 mmHg (node 15), and the SBP was below 188 mmHg (node 19), the probability of BP reduction with beta-blockers was 0.9 (terminal node 20, *p* = 0.048). When the DBP was below 105 mmHg (node 15), patients who received beta-blockers (BetaB = 1) had a probability of 0.7 to manage the treatment successfully when the SBP was above 180 mmHg (terminal node 18, *p* < 0.001). Two effective treatments from the beta-blocker class were identified. Metoprolol with a 0.9 probability of reducing BP to the selected interval when the SBP was above 181 mmHg (^**^, node 6), and the DBP was between 106 and 120 mmHg. Labetalol had a probability of 1 when the DBP was between 97 and 120 mmHg.

Patients who received EVT/tPA and were treated with vasodilators (DirectVasoD = 1, node 7) had a probability of 0.8 for predicting the primary outcome (terminal node 8, *p* < 0.001). However, the treatment of Hydralazine (^***^, node 7) was found to be less effective in predicting BP reduction to the selected interval (0.4 probability). Patients who received EVT/tPA had a very low probability of BP reduction when ACE-I (node 9), CCBs (node 11), or diuretics (node 14) were administered (*p* < 0.001). On the left side, patients who did not receive EVT/tPA with SBP above 220 mmHg (node 3) and those who received beta-blockers (node 4) had a probability of 0.8 for the prediction of the primary outcome (*p* < 0.001). The most significant treatment from the beta-blocker drug class was labetalol (^*^, node 4), with the same probability of BP reduction.

Figure 5A represents the left branch of the tree, where the MaxDBP is above 120 mmHg. On the left side, in patients who received beta-blockers (BetaB = 1, node 22), success in predicting the primary outcome was related to sodium levels (node 34, *p* = 0.01). For patients with SBP below or equal to 202 mmHg (node 33) and sodium levels higher than 129 mEq/L, using beta-blockers lowered the BP with a probability of 0.9 (terminal node 36). In comparison, for sodium values lower than 129 mEq/L, the probability of lowering the BP was 0.5 (terminal node 35), that is, random probability, and, thus, does not contribute to hypertension management.

The subtree for the beta-blocker class is shown in Figure 5B (^*^, node 22). When labetalol is administered, the probability of reducing BP is 0.9 (terminal node 38) if the SBP is below or equal to 200 mmHg. However, when BP is above 200 mmHg, the probability is 0.6 (terminal node 39). When metoprolol (node 40) is administered, the probability is 0.7 (terminal node 41).

On the right, patients who did not receive beta-blockers (BetaB=0, node 22) but received ACE inhibitors (node 29), direct vasodilators (node 23), and CCBs (node 31) had a probability of 1, 0.8, and 0.8, respectively (*p* < 0.001). Patients who received diuretics (node 24) had a high probability, 1, when the SBP was equal to or below 197 mmHg (node 26); however, when the SBP was above 197 mmHg, the probability of BP reduction was incredibly low and equal to 0.3 (*p* = 0.002).

As evident in Figure 5B, the most significant treatment from the ACE-I class is lisinopril (node 24, subtree ^***^), with the same probability of 1 to reduce BP to the interval. Amlodipine (node 30, subtree ^***^) was the most significant drug treatment from the CCB class and resulted in the reduction of BP with a higher probability (0.9). Furosemide (node 26, subtree ^***^) lowers BP with a probability of 0.8 (terminal node 22, subtree^***^) when the SBP (node 27) is below or equal to 204 mmHg. However, when the SBP is above 204 mmHg, the probability to lower BP to the selected interval is very low and equal to 0.1 (terminal node 29). Hydralazine (node 23, subtree^**^) lowers BP with a probability of 0.9 (terminal node 36) if the SBP is above 225 mmHg and lisinopril is not administered.

The decision trees presented in Figures 4, 5 yielded accuracy, F-score, and kappa values of 0.977, 0.884, and 0.871, respectively, for the original dataset and slightly lower values of 0.971, 0.857, and 0.841, respectively, for the reduced dataset, as shown in Table 3. The performance of the variant tree that yielded the subtrees in Figure 4 was similar to that of the original tree with accuracy, F-score, and kappa values of 0.971, 0.845, and 0.861, respectively.

**Table 3:** Comparison of the performances of the different models.

**Model**	**Original dataset**			**Dataset after feature selection**		
	**Accuracy**	**Kappa**	**F-score**	**Accuracy**	**Kappa**	**F-score**
Ctree	0.977	0.871	0.884	0.971	0.841	0.857
RandomForest	0.984	0.91	0.901	0.972	0.843	0.864
Logistic regression	0.93	0.52	0.557	0.928	0.531	0.569
Neural networks	0.963	0.769	0.789	0.968	0.822	0.840

**Figure 6:**
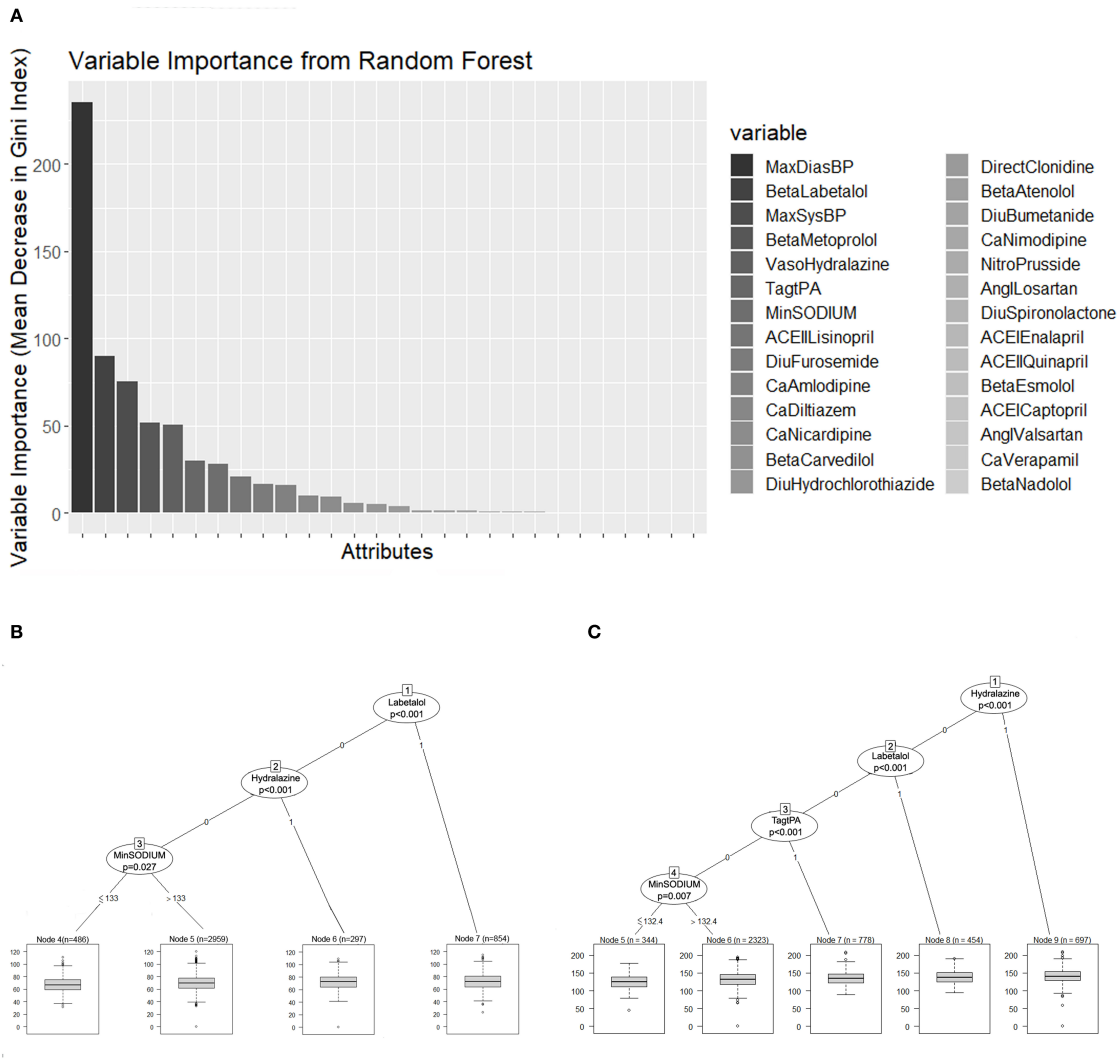
**(A)** Random forest variable importance by mean decrease in Gini index. **(B,C)** Represent the regression trees that predict the TWA of DBP and SBP, respectively. Each terminal node has a boxplot that shows the median, IQR, and outliers. TWA, time-weighted average; DBP, Diastolic blood pressure; SBP, systolic blood pressure; IQR, interquartile range.

We further evaluated the TWA of SBP and DBP with regression trees to assess the effect of the different antihypertensive drugs on BP reduction during the first 24 h of treatment. This additional assessment provided information regarding the level of BP achieved with the use of the different treatments. The diastolic (Figure 6B) and systolic BP values (Figure 6C) were evaluated independently as TWA over 24 hours. Only labetalol and hydralazine demonstrated a predictive association on regression analysis (*p* < 0.001).

Treatment with Labetalol predicted a TWA BP of 138.5/72.6 mmHg. When Hydralazine was used, the predicted TWA BP was 140.9/80 mmHg. The predicted TWA SBP for patients who received tPA was 134.7 mmHg.

Both regression trees showed a prediction of BP according to different sodium levels. When the sodium levels were higher than 132 mEq/L, the predicted TWA SBP was 131.6 mmHg, and for lower sodium levels, the predicted TWA SBP was 126 mmHg. When the sodium levels were higher than 133 mEq/L, the predicted TWA DBP was 70 mmHg, while for lower sodium levels, the predicted TWA DBP was 67.5 mmHg.

The RMSE of the TWA SBP regression tree model was 19.72 mmHg, and the MAE was 15.9 mmHg. Regarding the TWA DBP regression tree model, the RMSE was 24.6 mmHg, and the MAE was 10.22 mmHg.

### Classification Model for the Balanced Dataset

**Figure 7:**
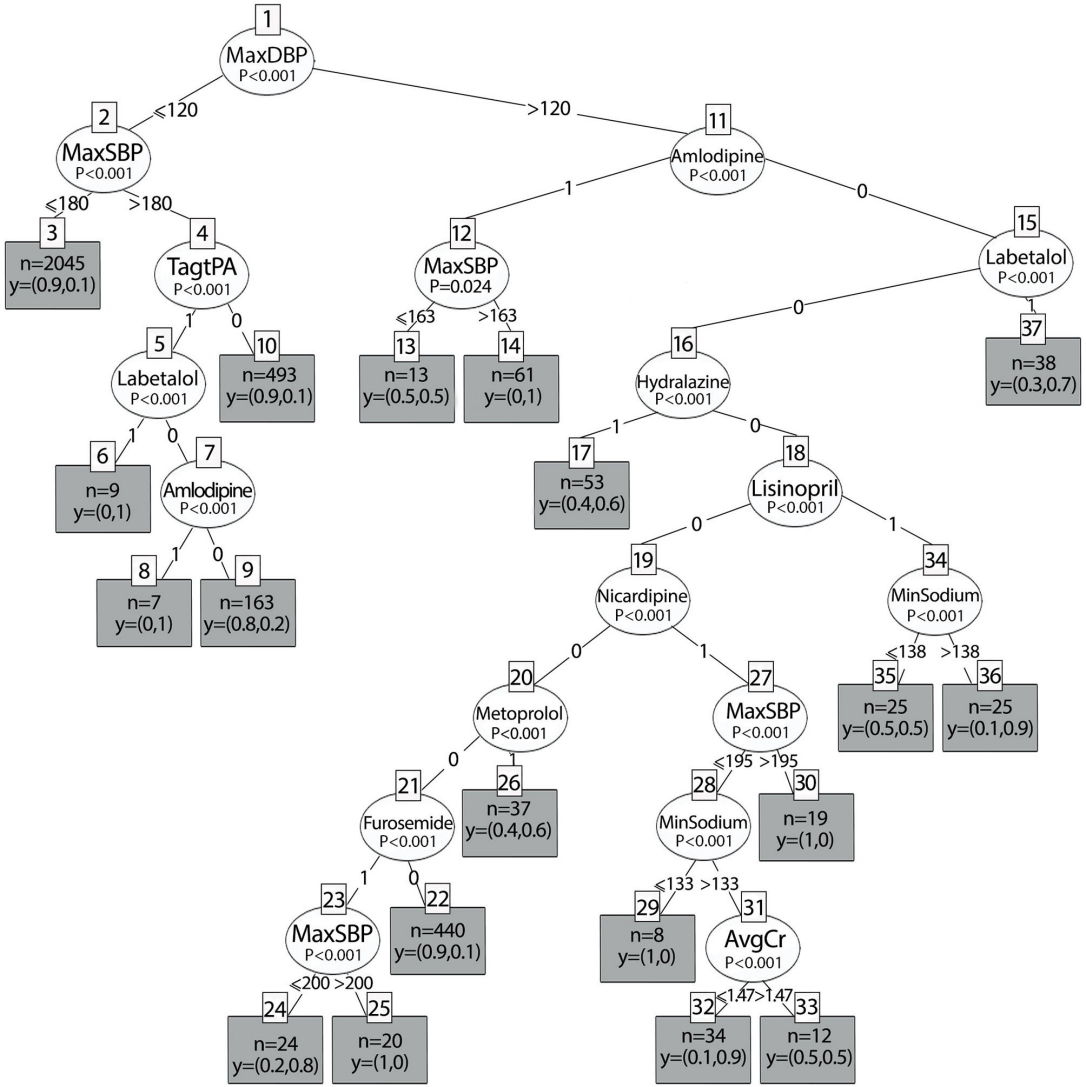
Decision tree results of the balanced dataset according to different antihypertensive treatments.

The results of the decision tree analysis based on the balanced dataset are shown in Figure 7. Similar to the imbalanced dataset, DBP was the main variable that predicted the primary outcome. In addition, when the maximum DBP is below 120 mmHg, BP management depends on the thrombolytic status. Of the eight medications that were selected to be included in the balanced dataset, only two medications in this group of patients who received tPA/EVT showed statistically significant results for lowering BP to the selected interval: Labetalol and Amlodipine. Both had a probability of 1 to lower BP, 10–30% of the maximum value in the first 24 h after stroke onset. This means that when a physician will treat patients who receive tPA/EVT according to guidelines (BP> 180/120 mmHg), he/she will lower the average BP until the end of the first 24 h by 10–30% of the maximum value if they will be treated with labetalol or amlodipine. When the DBP is above 120 mmHg, the probability of lowering BP depends on the SBP and on the specific treatment that was administered. In addition, BP reduction also depends on the sodium level and kidney function (creatinine levels). Accordingly, when the BP is above 163/120 mmHg, the probability of lowering BP with amlodipine was 1, whereas when the SBP was below 163 mmHg, the probability of decreasing BP to the interval was only 0.5, that is, a random probability (*p* = 0.024, terminal nodes 14 and 13, respectively). When Labetalol is administered, the probability is 0.7 (*p* < 0.001, terminal node 37). This means that in high levels of BPs, there is a 70% probability that BP will be lowered by Labetalol to the selected interval, but in the other 30%, whether BP is decreased is unknown. When Hydralazine is administered, the probability is 0.6 (*p* < 0.001, terminal node 17). When lisinopril is administered, the probability of lowering BP to the interval depends on the sodium levels: above 138 mEq/L, the probability is 0.9, while for lower sodium levels, the probability is much lower and equals 0.5. The probability of lowering BPs with nicardipine depends on the BP levels, kidney function, and sodium levels, and accordingly, is equal to 0.9 when the SBP is below 195 mmHg, the creatinine levels are below 1.47 mg/dL, and the sodium levels are 133 mEq/L. However, when the sodium levels are lower, the probability of lowering the BP to the interval is 0. The probability of lowering BP when the DBP is below 120 mmHg with metoprolol is low and equals 0.6 (*p* < 0.001, node 26). The probability of lowering BP with furosemide depends on the SBP levels. When the SBP was above 200 mmHg, the probability was 0.83. However, for SBP <200 mmHg, the probability was 0 (*p* < 0.001, terminal nodes 25 and 26, respectively). The performance of the balanced tree yielded high accuracy, F-score, and kappa values of 0.94, 0.8, and 0.75, respectively.

### Random Forest Model

Random forest algorithms aggregate many decision trees and add randomness to the model, thus, improving the performance of the decision trees and reducing overfitting (42). The classification random forest algorithm was implemented using the R package *randomForest* (43). It was tuned with a random search of the number of variable samples at each split using *the caret* package in R (38).

Each decision tree was randomly selected from a given dataset using different bootstrap samples. The random forest algorithm obtains a prediction from each tree, performs a vote for each predicted result, and then selects the best solution with the majority of votes. The most important variables used in the algorithm are those with a lower probability of incorrect classification.

Figure 6A represents the random forest variable importance based on the mean decrease in the Gini index. MaxDBP, Labetalol, MaxSBP, Metoprolol, Hydralazine, Tag tPA, and minimum sodium levels are the most important variables in the prediction task, similar to the results represented by the decision trees.

### Neural Networks

**Figure 8:**
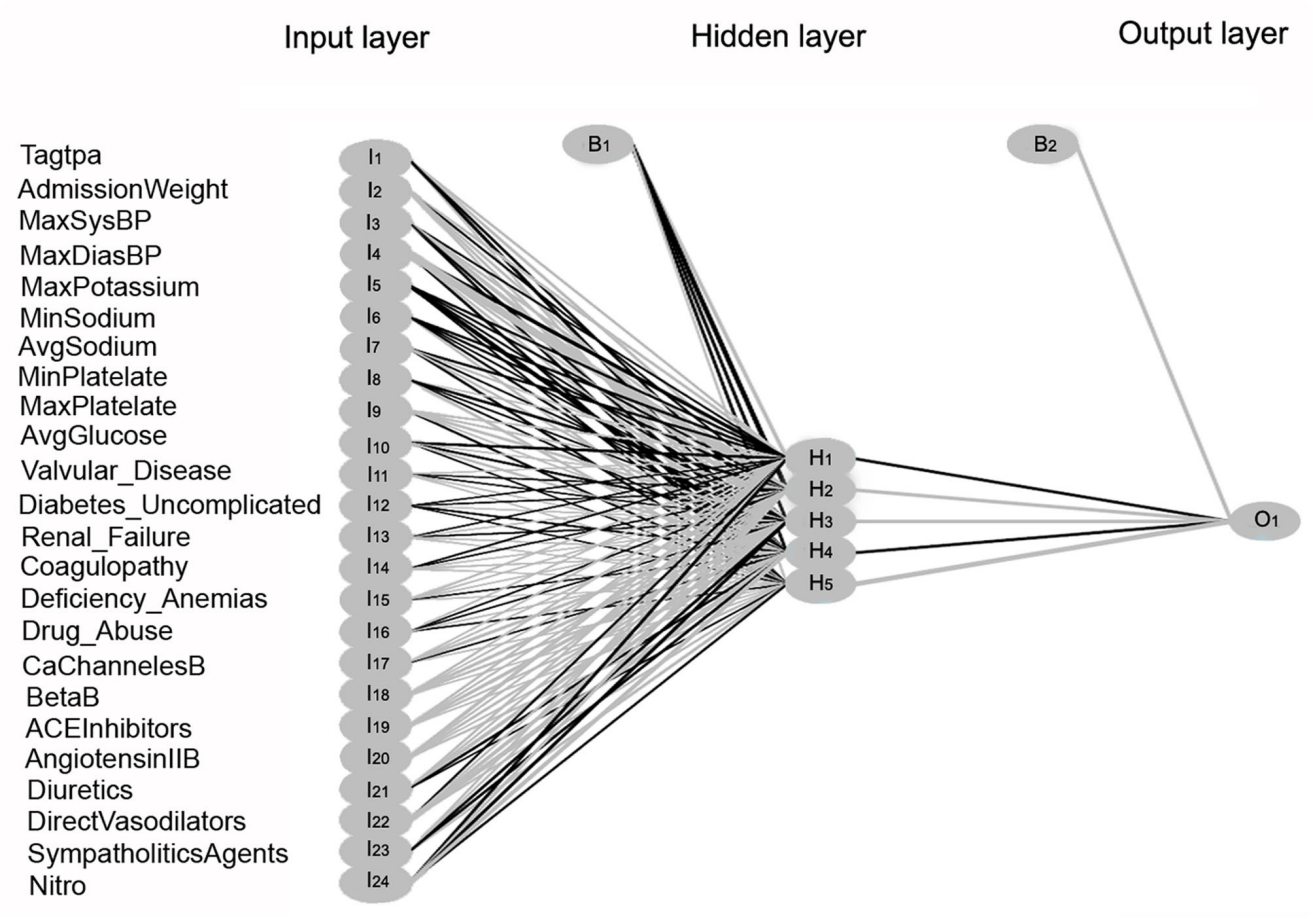
Multi-Layer Perceptron neural network model illustration. Three layers within the neural network are shown: the input layer, hidden layer, and output layer. Twenty-four input variables labeled as I1-I24 are transmitted to the hidden layer. Within the hidden layer, neuron-like units are labeled as H1-H5. The bias nodes are labeled as B1 and B2. The response variable in the output layer is labeled as O1.

The feed-forward multi-layer perceptron (MLP) neural network algorithm was implemented using the R package *nnet* (36). As shown in Figure 8, 24 input variables were received in the first layer on the left and processed within a hidden intermediate layer, using a weighted summation and an activation function. Within the hidden layer, a learning algorithm optimizes the weight between two connected neuron-like units. The bias nodes shift the activation function and generate better prediction results. The output layer on the right produces the result for a given input from the hidden layer.

Comparing the different classification models, the highest performance was found for the random forest model with accuracy, kappa, and F-score values of 0.984, 0.91, and 0.901, respectively, for the original dataset, and 0.972, 0.843, and 0.864, respectively, for the reduced dataset. The results of the performance of the different models are listed in Table 3.

The moderate model performance of the logistic regression model is shown by lower accuracy, kappa, and F-score values of 0.93, 0.52, and 0.557, respectively, for the original dataset, and 0.928, 0.531, and 0.569, for the reduced dataset. After the random forest and decision tree, the neural network algorithm showed high accuracy, F-score, and kappa values.

## Discussion

This study assesses whether a patient who receives antihypertensive treatment, according to recommended thresholds, is an effective treatment that lowers BP in the range of 10–30% below the maximum BP value in the first 24 h after the acute ischemic stroke onset. In comparison to randomized clinical trials that were tested in conventional methods, one or two medications in each clinical trial and compare the results to a placebo group, our study simultaneously examined over 100 antihypertensive medications using ML techniques.

This study has three major findings:

1. Blood pressure (BP) management in the first 24 h after a stroke should be managed according to different BP levels and other clinical variables, mainly kidney functioning and sodium levels. Diastolic blood pressure (DBP) is the main variable predicting the probability of BP reduction in the first 24 h after acute ischemic stroke.
2. For patients receiving tPA/EVT, labetalol and amlodipine are effective treatments when antihypertensive treatment is administered in cases where SBP > 180 mmHg and DBP <120 mmHg.
3. Monitoring and treating low sodium levels (<133 mEq/L) is important for successful BP reduction in the first 24 h after the acute ischemic stroke in patients with very high BP (DBP> 120 mmHg). The independent regression analysis predicting the TWA BP supports the relationship between sodium levels and BP.

In accordance with the AHA/ASA recommendations, we found that the group of patients receiving tPA would benefit from beta-blockers when DBP ranges from 105 to 120 mmHg with a high probability of reducing BP to the selected interval with Labetalol (*p* = 0.004). These results are supported by the analysis of both balanced and imbalanced datasets. Labetalol also lowers BP with a probability ranging between 0.6 and 0.7 when the BP is above 200/120 mmHg, that is, in 30–40% of cases, and the goal of BP reduction to the interval is not reached. This may reflect the difficulty of lowering BP with labetalol in patients with excessively high BP. Treatment with labetalol predicted a TWA BP of 138.5/72.6 mmHg (*p* < 0.001) in the hyper-acute phase after a stroke. Unlike current recommendations, patients receiving EVT/tPA with DBP <120 mmHg had a low probability of BP reduction over the prescribed range with CCB therapy (*p* < 0.001) as a class group. However, the balanced dataset analysis showed very good results with the use of amlodipine. The use of amlodipine for patients receiving tPA/EVT should be further studied as a potential antihypertensive treatment in this group of patients. This is a novel finding that emerged from this study and has practical importance.

Another important finding is the significance of electrolyte balance, especially sodium levels, in BP management. In patients with DBP above 120 mmHg, successful BP management is related to sodium levels. According to the balanced results, both lisinopril and nicardipine treatments depend on avoiding hyponatremia. Sodium levels lower than 129–133 mEq/L do not contribute to hypertension management in acute stroke because the probability of BP reduction is 0–0.5. Nicardipine is also effective only when kidney functioning is normal or only slightly elevated (creatinine <1.47 mg/dl) and when the SBP is below 195 mmHg. Above an SBP of 195 mmHg, the probability of lowering BP to the interval with nicardipine was very low. However, we cannot exclude a temporary response to a lower BP with nicardipine. According to the analysis of the imbalanced dataset, nicardipine was not included in the results of the decision tree algorithms. This might have resulted from the relatively lower number of observations with nicardipine treatment. The balanced dataset corrected the bias of minority representation of nicardipine, which can be attributed to different protocols that were used in different ICU units and found to be statistically significant for the prediction task. Similar results for the regression trees indicated a significant relationship between BP and sodium levels. The regression trees predicted higher DBP and SBP for higher sodium levels above 132 mEq/L and a modest reduction in BP of 5.6/2.5 mmHg (*p* < 0.05) for lower levels of sodium.

Hyponatremia is common in patients with acute stroke and is attributed to stroke-related causes, such as elevated secretion of antidiuretic hormone (ADH) and salt-wasting syndrome, as well as to non-stroke-related causes, such as comorbidities, the use of certain medications, and iatrogenic causes (44). The different mechanisms by which hyponatremia occurs in acute stroke patients might account for the difficulty in predicting the success of the BP-lowering strategy in patients with hyponatremia. The relationship between sodium levels and BP during the acute phase of ischemic stroke is not well-established; however, in certain circumstances, hyponatremia is related to fluid imbalance and might impair BP regulation (45).

In accordance with the AHA/ASA recommendations to use CCBs (nircadipine, clevidipine) and the INWEST trial that showed a significant decrease in SBP and DBP with nimodipine (18), we found that nircadipine and amlodipine are efficient in lowering BP to the target interval under certain conditions. Regarding amlodipine, when BP is above 163/120 mmHg, the probability of lowering BP ranges between 0.9 and 1. In patients receiving tPA/EVT with DBP <120 mmHg, only amlodipine was found to be effective. As discussed above, treatment with nicardipine is effective when SBP <195 mmHg, and success depends on sodium and creatinine levels.

Similar to the CHHIPS trial that showed a significant decrease with lisinopril (16), our results show that patients with DBP > 120 mmHg who received lisinopril had a high probability (0.9–1) to lower the BP to the target interval, with a statistical significance of *p* < 0.001. These results are supported by the analysis of both balanced and imbalanced datasets. According to the imbalanced dataset, Lisinopril showed a low probability of reducing BP when the DBP was lower than 120 mmHg. Treatment with lisinopril was not included in the results of the balanced dataset for DBP <120 mmHg (right branch of the tree, Figure 6), because it did not meet the minimum criteria of split *p* < 0.05, or because other variables (selected by the decision tree algorithm) had a lower *p*-value and contributed more to the prediction task.

When DBP was above 120 mmHg, both hydralazine and metoprolol showed a low probability of lowering BP (0.6). The imbalanced dataset showed that treatment with Hydralazine was effective when SBP was above 225 mmHg. However, for lower BPs, hydralazine lowered BP with a very low probability. This is not reflected in the balanced dataset analysis. This might result from the fact that the imbalanced dataset also included patients who received more than one antihypertensive treatment. Therefore, we can see that subtree^**^ in Figure 5 includes more than one medication, and part of the results depend on administering hydralazine with lisinopril as an adjuvant treatment. Metoprolol lowers BP with a probability of 0.6–0.7 when the DBP is above 120 mmHg. When the DBP is between 106 and 120 mmHg, the probability of lowering BP to the interval is high and equals 0.9, according to the imbalanced analysis. As with lisinopril, metoprolol was not included in the analysis of the imbalanced dataset when the DBP was below 120 mmHg.

The algorithm shows that BP reduction with furosemide is effective when the SBP is below 200 mmHg with a high probability, 0.8, which will reduce BP. However, in patients with SBP > 200 mmHg, the probability of lowering BP is very low. This might be related to the fact that furosemide inhibits the Na-K-Cl cotransporter, resulting in the increased secretion of sodium in the urine. This explanation is suggested because the results indicate that effective treatment depends on avoiding low sodium levels for certain medications (nicardipine and lisinopril).

We emphasize that the analysis with ML techniques to predict outcomes indicates the relationships between predictors and outcomes rather than causality.

In our research, ARBs were not included in the decision tree results because the variable did not reach the minimum criterion of *p* < 0.05. These results are consistent with the trials that showed a significant decrease of <10/6 mmHg when compared to placebo (19, 20). This decrease is likely not to exceed a threshold of a 10% reduction in BP.

We found that both decision tree (Ctree) and random forest algorithms have very high accuracy, kappa, and F-score values. We chose a subset of features by using a bidirectional stepwise algorithm that selected the most relevant features from the original dataset and, thus, decreased the model complexity without significantly reducing the prediction accuracy. The high kappa and F-score values indicate the high validity of the models. The moderate model performance of the logistic regression model vs. the decision tree algorithm is evident by the lower kappa and F-score values. Similar results of better accuracy performance when tree algorithms were compared to logistic regression were found when tested on large datasets (46). The neural network algorithm also showed high accuracy, F-score, and kappa values, but these values were slightly lower than those of the decision tree and random forest algorithms. However, the neural-network algorithm showed better performance after the feature selection process. Nonetheless, it is difficult to visually represent and explain neural networks.

### Limitations

This study has several limitations. Our study reflects the current general recommendations and current practices for hypertension management after acute stroke. However, the two databases that were used included data that were collected during 2001–2012 (MIMIC-III) and 2014–2015 (eICU) and may reflect the management of stroke patients before the up-to-date recommendations that replaced the 2013 guidelines for the early BP management of patients with acute ischemic stroke. In addition, the exact range of BP lowering in extremely high BP is not well-established yet. Patients with severe hypertension (> 220/120 mmHg) were usually excluded from studies that examined the clinical outcomes related to BP lowering after acute ischemic stroke. Thus, whether a lowering of 10–30% from the maximum BP for highly elevated BP is too drastic should be further studied. Another important issue that clinicians should take into consideration is that the decision-making in hypertension management after acute stroke should not guide treatment alone, and clinical judgment is cardinal, especially for patients with acute concomitant comorbidities (such as acute myocardial infarction, acute heart failure, aortic dissection, or preeclampsia/eclampsia) in whom treatment should be individualized. In addition, it is important to pay attention to the contraindications of certain antihypertensive drug classes. The use of beta-blockers in acute decompensation of heart failure or the use of beta-blockers in patients with asthma exacerbation are some of the examples. Further research is needed to examine the outcomes of BP lowering in the acute phase after ischemic stroke, especially in patients with severe hypertension (>220/120), who are underrepresented in clinical trials. Outcomes according to different BP-lowering intervals should be further examined.

## Conclusion

This is the first study to address BP management in the acute phase following ischemic stroke using ML techniques. The study shows that the choice of antihypertensive treatment in the context of acute ischemic stroke should be adjusted to different BP levels and clinical features of the patient, thus providing a better decision-making approach. Further work will clarify whether there are different subgroups of patients for whom specific BP management options are better, and might include additional outcomes such as morbidity levels, mortality, readmission to hospitals, and recurrent stroke. ML techniques are used to discover hidden patterns from data and to apply robust interrogation to datasets; however, there is a risk of overfitting. However, the potential improvement in BP management in acute ischemic stroke suggested in this study should not be ignored. Rather, follow-up studies should further examine the strategies to reduce inherent ML risks and attempt to replicate the results of clinical studies.

## Data Availability Statement

Publicly available datasets were analyzed in this study. This data can be found at: Johnson, A., Pollard, T., & Mark, R. (2019). MIMIC-III Clinical Database Demo (version 1.4). PhysioNet. https://doi.org/10.13026/C2HM2Q. The eICU Collaborative Research Database, a freely available multi-center database for critical care research. Pollard TJ, Johnson AEW, Raffa JD, Celi LA, Mark RG, and Badawi O. Scientific Data (2018). http://dx.doi.org/10.1038/sdata.2018.178. Available from: https://www.nature.com/articles/sdata2018178.

## Ethics Statement

MIMIC-III and eICU databases have received ethical approval from the Institutional Review Boards (IRBs) at BIDMC and MIT and because the database does not contain protected health information, a waiver for the requirement for informed consent was included in the IRB approval. Researchers using those databases are required to formally request access. There are two key steps that must be completed before access is granted: the researcher must complete a recognized course in protecting human research participants that includes Health Insurance Portability and Accountability Act (HIPAA) requirements. The researcher must sign a data use agreement, which outlines appropriate data usage and security standards, and forbids efforts to identify individual patients. Access was granted to OM on July 28, 2019. Written informed consent for participation was not required for this study in accordance with the national legislation and the institutional requirements.

## Author Contributions

All authors made substantial contributions to the conception, design of the work, contributed to revising it critically for important intellectual content, and gave their final approval of the version to be published. OM was also responsible for the extraction, analysis of the data, and manuscript drafting.

## Conflict of Interest

The authors declare that the research was conducted in the absence of any commercial or financial relationships that could be construed as a potential conflict of interest.

## Publisher's Note

All claims expressed in this article are solely those of the authors and do not necessarily represent those of their affiliated organizations, or those of the publisher, the editors and the reviewers. Any product that may be evaluated in this article, or claim that may be made by its manufacturer, is not guaranteed or endorsed by the publisher.
