# Supplementary material for: Machine Learning Techniques in Blood Pressure Management During the Acute Phase of Ischemic Stroke
Source: Front Neurol. 2022 Feb 14;12:743728. doi: 10.3389/fneur.2021.743728 (PMC8882601; doi:10.3389/fneur.2021.743728)
Supplement: Supplementary file 1 [file Table_1.DOCX]

**SUPPLEMENTAL MATERIAL**

**Supplemental Tables**

**Table I.** **ICD9 diagnosis codes for acute ischemic stroke**

| ICD9 codes | Description | | | Not received  EVT/tPA | | Received  EVT/tPA | |
| --- | --- | --- | --- | --- | --- | --- | --- |
|  | |  | Number of patients | | Number of  Icustays | Number of patients | Number of  Icustays |
| 433.01 | Occlusion and stenosis of basilar artery with cerebral infarction | | | 16 | 16 | 11 | 11 |
| 433.11 | Occlusion and stenosis of the carotid artery with cerebral infarction | | | 39 | 40 | 22 | 22 |
| 433.21 | Occlusion and stenosis of vertebral artery with cerebral infarction | | | 12 | 12 | 0 | 0 |
| 433.31 | Occlusion and stenosis of multiple and bilateral precerebral arteries with cerebral infarction | | | 16 | 16 | 7 | 7 |
| 433.81 | Occlusion and stenosis of other specified precerebral artery with cerebral infarction | | | 2 | 2 | 0 | 0 |
| 433.91 | Occlusion and stenosis of unspecified precerebral artery with cerebral infarction | | | 1 | 1 | 1 | 1 |
| 434.00 | Cerebral thrombosis without mention of cerebral infraction | | | 2 | 2 | 0 | 0 |
| 434.10 | Cerebral embolism without mention of cerebral infraction | | | 1 | 1 | 0 | 0 |
| 434.90 | Cerebral artery occlusion, unspecified without mention of cerebral infarction | | | 14 | 14 | 0 | 0 |
| 434.01 | Cerebral thrombosis with cerebral infarction | | | 21 | 21 | 19 | 19 |
| 434.11 | Cerebral embolism with cerebral infarction | | | 474 | 524 | 140 | 142 |
| 434.91 | Cerebral artery occlusion, unspecified with cerebral infarction | | | 2733 | 2996 | 979 | 1017 |
| 436 | CVA, Acute, but ill-defined, cerebrovascular disease | | | 2859 | 3132 | 448 | 457 |
| Total |  | | | 6190 | 6777 | 1627 | 1676 |

Notes; EVT: Endovascular therapy, tPA: tissue plasminogen activator, CVA: Cerebrovascular Accident.

**Table II. Variables Description and rate of missing values**

|  | Variable | Description | Missing rates (%) |
| --- | --- | --- | --- |
| Demographics | Gender | Female or male | 0 |
|  | Ethnicity | Patient ethnic group | 0 |
|  | Age | Patient age | 0 |
|  | Hight | Patient height | 11.57 |
|  | Weight | Patient weight | 10.19 |
| Hemodynamic | Systolic BP | Systolic blood pressure | 2.28 |
| Variables | Diastolic BP | Diastolic blood pressure | 2.28 |
| and vital signs | Mean BP | The average blood pressure | 2.28 |
|  | CI | Cardiac index: cardiac output from the left ventricle to surface body area per minute | 0 |
|  | CVP | Central Venous Pressure | 0 |
|  | Heart Rate | Number of contractions of the heart per minute | 1.29 |
|  | SpO2 | Oxygen saturation | 2.07 |
|  | Respiratory Rate | Breaths per minute | 6.55 |
|  | Ventilation Tag | Patients on mechanical ventilation | 0 |
|  | tPATag | Patients who received endovascular treatment | 0 |
| Lab results | Bicarbonate | Bicarbonate in blood | 12.29 |
|  | PH | Blood PH | 14.24 |
|  | Glucose | The glucose level in the blood | 4.224 |
|  | Cr | Creatinine level in the blood | 7.50 |
|  | BUN | Blood urea nitrogen | 7.68 |
|  | K | Potassium level in the blood | 7.66 |
|  | Na | The sodium level in the blood | 7.44 |
|  | Mg | Magnesium level | 1.16 |
|  | P | Phosphate level | 1.91 |
|  | Ca | Calcium total level | 9.47 |
|  | Cl | Chloride level | 7.97 |
|  | Hemoglobin | Hemoglobin in blood | 8.36 |
|  | Hematocrit | Hematocrit in blood | 7.91 |
|  | MCV | Mean corpuscular volume blood test | 10.99 |
|  | MCH | Mean cell Hemoglobin | 10.75 |
|  | MCHC | mean corpuscular hemoglobin concentration blood test | 10.75 |
|  | RDW | Red cell distribution width blood test | 14.11 |
|  | PLT | Platelets count in the blood | 9.17 |
|  | WBC | White blood cells in the blood | 8.29 |
| History | CHF | Congestive heart failure | 0 |
|  | Cardiac arrhythmias | Heart rhythm problems | 0 |
|  | Valvular disease | Valvular heart disease | 0 |
|  | Pulmonary circulation | Pulmonary circulation disorders | 0 |
|  | Peripheral vascular | Peripheral vascular disorders | 0 |
|  | HTN | Hypertension | 0 |
|  | Chronic pulmonary | Chronic pulmonary disease | 0 |
|  | Diabetes uncomplicated | Patients with uncomplicated diabetes | 0 |
|  | Diabetes complicated | Patients with complicated diabetes | 0 |
|  | Hypothyroidism | Low thyroid disorder | 0 |
|  | Renal failure | Kidney failure | 0 |
|  | Liver disease | Hepatic disorders | 0 |
|  | Peptic ulcer | Peptic ulcer disease | 0 |
|  | AIDS | Acquired immunodeficiency syndrome | 0 |
|  | Lymphoma | Blood cancers that develop from lymphocytes` | 0 |
|  | Metastatic cancer | Advanced metastatic cancer | 0 |
|  | Solid tumor | Solid tumor without metastasis | 0 |
|  | RA | Rheumatoid arthritis or collagen vascular diseases | 0 |
|  | Coagulopathy | Bleeding disorder | 0 |
|  | Obesity | Obese patients | 0 |
|  | Weightless | Reduction of the total body mass | 0 |
|  | Alcohol abuse | Alcohol abuse | 0 |
|  | Drug abuse | Substance or drug use disorder | 0 |
|  | Psychosis | Psychotic disorders | 0 |
|  | Depression | Depressive mood disorder | 0 |

**Table III. Antihypertensive medications that were extracted from the API interface**

| Class | Drugs that were  Extracted |
| --- | --- |
| CCBs | Amlodipine, Clevidipine, Diltiazem, Felodipine, Isradipine, Lacidipine, Lercanidipine, Manidipine, Mepirodipine, Nicardipine, Nifedipine, Nilvadipine, Nimodipine, Nisoldipine, Nitrendipine, Verapamil |
| Beta blocking agents | Acebutolol, Alprenolol, Atenolol, Atenolol-nifedipine, Betaxolol Bisoprolol, Bopindolol, Bupranolol, Carvedilol ,Celiprolol , Esmolol, Felodipine-metoprolol, Labetalol, Metoprolol ,Nadolol ,Nebivolol, Oxprenolol ,Penbutolol , Pindolol , Propranolol, Tertatolol |
| ACE-inhibitors | Captopril, Enalapril, Benazepril, Cilazapril, Fosinopril, Imidapril, Lisinopril, Moexipril, Perindopril, Quinapril, Ramipril, Spirapril, Trandolapril, Zofenopril |
| ARBs | Azilsartan-medoxomil, Candesartan, Eprosartan Irbesartan, Losartan, Olmesartan, Medoxomil, Telmisartan, Valsartan |
| Diuretics | Amiloride, Bendroflumethiazide, Bendroflumethiazide-Potassium Bumetanide, Bumetanide-Potassium, Canrenoate, Chlorothiazide, Chlorthalidone, Cicletanine, Clopamide, Cyclopenthiazide, Cyclothiazide, Eplerenone, Ethacrynic-Acid, Furosemide, Furosemide-Potassium, Hydrochlorothiazide, Hydroflumethiazide, Indapamide, Mefruside, Methyclothiazide, Metolazone, Piretanide, Polythiazide, Quinethazone, Spironolactone, Torsemide, Triamterene, Trichlormethiazide, Xipamide |
| Direct vasodilators | Hydralazine, Dihydralazine, Minoxidil |
| Sympatholytic agent | Clonidine, Methyldopa, Reserpine, Guanfacine |
| Other | Nitroprusside |

Notes; CCBs: Calcium channels blockers, ACE: Angiotensin-converting enzyme, ARBs: Angiotensin receptor blockers.
